# The Association between Individual Income and Aggressive End-of-Life Treatment in Older Cancer Decedents in Taiwan

**DOI:** 10.1371/journal.pone.0116913

**Published:** 2015-01-13

**Authors:** Chih-Yuan Huang, Yeh-Ting Hung, Chun-Ming Chang, Shiun-Yang Juang, Ching-Chih Lee

**Affiliations:** 1 Division of Nephrology, Department of Internal Medicine, Dalin Tzu Chi Hospital, Buddhist Tzu Chi Medical Foundation, Chiayi, Taiwan; 2 Department of Pediatrics, Dalin Tzu Chi Hospital, Buddhist Tzu Chi Medical Foundation, Chiayi, Taiwan; 3 Department of Surgery, Dalin Tzu Chi Hospital, Buddhist Tzu Chi Medical Foundation, Chiayi, Taiwan; 4 Department of Medical Research, Dalin Tzu Chi Hospital, Buddhist Tzu Chi Medical Foundation, Chiayi, Taiwan; 5 School of Medicine, Tzu Chi University, Hualian, Taiwan; 6 Department of Otolaryngology, Dalin Tzu Chi Hospital, Buddhist Tzu Chi Medical Foundation, Chiayi, Taiwan; 7 Center for Clinical Epidemiology and Biostatistics, Dalin Tzu Chi Hospital, Buddhist Tzu Chi Medical Foundation, Chiayi, Taiwan; 8 Center for Clinical Epidemiology and Biostatistics, Dalin Tzu Chi Hospital, Buddhist Tzu Chi Medical Foundation, No.2, Ming-Sheng Road, Dalin Town, Chiayi, 622, Taiwan (ROC); University of Pennsylvania, UNITED STATES

## Abstract

**Objectives:**

To examine the association of individual income and end of life (EOL) care in older cancer decedents in Taiwan.

**Design:**

Retrospective cohort study.

**Setting:**

National Health Insurance Research Database (NHIRD) in Taiwan.

**Participants:**

28,978 decedents >65 years were diagnosed with cancer and died during 2009-2011 in Taiwan. Of these decedents, 10941, 16535, and 1502 were categorized by individual income as having low, moderate, and high SES, respectively.

**Main outcome measures:**

Indicators of aggressiveness of EOL care: chemotherapy use before EOL, more than one emergency department (ER) visit, more than one hospital admission, hospital length of stay >14 days, intensive care unit (ICU) admission, and dying in a hospital.

**Results:**

Low individual income was associated with more aggressive EOL treatment (estimate -0.30 for moderate income, -0.27 for high income, both p<0.01). The major source of aggressiveness was the tendency for older decedents with low income to die in the acute care hospital. The indicators had an increasing trend from 2009 to 2011, except for hospital stay >14 days.

**Conclusions:**

Low individual income is associated with more aggressive EOL treatment in older cancer decedents. Public health providers should make available appropriate education and hospice resources to these decedents and their families, to reduce the amount of aggressive terminal care such decedents receive.

## INTRODUCTION

Cancer has been the leading cause of death in Taiwan for decades [1]. Decedents older than 65 years account for 47.1% of new cancer cases, and 59.8% of cancer deaths [2]. Globally, an estimated 12.7 million new cancer cases and 7.6 million cancer deaths occurred in 2008 [3]. End-of-life (EOL) care is an issue in terminal decedents with cancer, with more aggressive care requiring greater healthcare spending in Taiwan over the last decade [4, 5]. In the United States, treatment for decedents in their last year of life accounted for more than one-quarter of Medicare spending [6]. In Canada, decedents in the final six months of life comprised 1.1% of the population but consumed 21.3% of health care [7]. Thus, evaluating the aggressiveness of EOL care in terminal cancer decedents and defining the determinants of such overuse of care are important for older Taiwanese with cancer, both medically and financially.

Data do not agree about the impact of socioeconomic status (SES) on aggressiveness of EOL care. Some studies show weak negative trends between EOL spending and area level income [8, 9]. Others show a positive association of higher SES with EOL spending [10–12].

Earle et al. has developed a set of indicators to evaluate aggressiveness of EOL care using administrative data [13]. Using the National Health Insurance Research Database (NHIRD), this study explored the association of indictors for aggressive EOL care with SES for older cancer decedents in Taiwan.

## METHODS

### Study Design and Sample

**Database.** The data for this study were collected from the Taiwan NHIRD for the years 2009 to 2011. This dataset is organized and managed by the Taiwan National Health Research Institutes but collected by the Taiwan National Health Insurance Program, in place since 1995. Taiwan’s NHI has the unique characteristics of universal insurance coverage, comprehensive services provided, and a single-payer system with the government as sole insurer. Patients have free access to any healthcare facilities they choose. Healthcare systems are reimbursed from Taiwan’s National Health Insurance Administration Ministry of Health and Welfare for services they provided. The program covers approximately 99% of the residents in Taiwan and has contracts with 97% of medical providers nationally. To verify the accuracy of diagnosis, the Taiwan Bureau of National Health Insurance randomly reviews the charts of one per 100 ambulatory and one per 20 inpatient claims[14]. All patient data were reviewed retrospectively.

Our study cohort consisted of older adult decedents (age > 65 years) with cancer as identified by the International Classification of Diseases, Ninth Revision, Clinical Modification [ICD-9-CM]. Diagnosis was verified by using the catastrophic illness dataset. Decedents also had a record of death during the study period (2009–2011).

### Measurement

**Aggressiveness of EOL care.** This study measured the aggressiveness of EOL care as a dependent variable using the following six quality indicators in the last month of life suggested by Earle et al. [13]: chemotherapy use before EOL, more than one emergency department (ER) visit, more than one hospital admission, hospital length of stay >14 days, intensive care unit (ICU) admission, and dying in a hospital. These data were collected from the NHIRD dataset within one month of death. Aggressiveness of EOL care was evaluated by assigning each patient a composite score which was the summation of all indicators. This composite score ranged from 0 to 6, with higher scores indicating more aggressive EOL care [15].

**Individual SES.** The four-factor Hollingshead scale uses marital status, gender, education and occupation [16]. Because other factors, such as marital status, and education level can’t be extracted from the NHIRD, this study used income-related insurance payment amount as a proxy measure of individual SES, which is an important prognostic factor for cancer [17, 18]. This method had been validated in several studies [19, 20]. The older decedents with cancer diagnosis were classified into three groups: (1) low SES, lower than US $528 per month (New Taiwan Dollars (NT) $1 to $15,840), (2) moderate SES, between US$528 to $833 per month (NT $15,841 to $25,000), and (3) high SES, US$833 per month (NT $25,001) or more [17]. We selected NT$15,840 as the low income level cutoff point because this was the government stipulated minimum wage for full-time employees in Taiwan in 2006.

**Patient characteristics.** Patient characteristics were recorded, including age, gender, urbanization level, geographic region, disease severity, post-diagnosis survival duration, cancer diagnosis, and primary physician’s specialty. Disease severity was estimated by using the Deyo adaptation of the Charlson Comorbidities Index Score (CCIS), which was derived from inpatient diagnoses in the last six months of life [21, 22]. Diagnosis and metastatic status were combined to identify seven subgroups (I-VII) of cancers that were homogeneous in terms of survival and disease course [15]. Metastatic status was identified by using ICD-9 codes 196.xx to 199.xx. Subgroups included four cancer types: germ cell tumors and prostate cancer; lung, liver, and pancreatic cancer; hematologic malignancies; and all other cancers. Survival time was calculated as the interval (in months) between the date of diagnosis and death, then categorized into 1–2, 3–6, 7–12, 13–24, and 25 or more months. The primary physician’s specialty was retrieved from the code in National Health Insurance claims and was divided into oncologist and other. Hospital characteristics such as accreditation level, case load, urbanization level, and geographic region were recorded.

### Statistical analysis

All data were analyzed using SPSS (version 15, SPSS Inc., Chicago, IL). Pearson’s chi-square test was used for categorical variables such as gender, level of urbanization, geographic region of residence, CCIS category, cancer group, and hospital characteristics (teaching level, geographic region, and caseload). Continuous variables were analyzed using one-way ANOVA.

The impact of each explanatory variable on the aggressiveness of EOL care was examined by hierarchical linear regression using a random-intercept model. A multilevel logistic regression model was constructed to explore the association of SES category with each indicator of aggressive EOL care after adjusting for patient characteristics (age, gender, cancer type, post-diagnosis survival, CCIS score, urbanization and geographic area, primary physician specialty, and hospital characteristics. A p-value of *P*<0.05 was used to indicate statistical significance.

### Ethics statement

The Institutional Review Board of Dalin Tzu Chi Hospital, Taiwan approved this study. Review board requirements for written informed consent were waived because all personal identifying information was removed from the NHIRD database prior to data analysis.

## RESULTS

A total of 28,978 terminal cancer decedents from 2009 to 2011 were identified. Of these, 10941, 16535, and 1502 were categorized as having low, moderate, and high income, respectively. Their basic characteristics are described in Table 1.

**Table 1:** Baseline characteristics of older patients (age >65 years) in Taiwan with terminal cancer by years (2009–2011) and total.

			**Socioeconomic status**						
**Parameter**	**Total**		**Low**		**Moderate**		**High**		*p* **value**
	**No.**	**%**	**No.**	**%**	**No.**	**%**	**No.**	**%**	
Total	28978	100	10941	37.8	16535	57.1	1502	5.2	
Gender									<0.001
Female	8770	30.3	2269	20.7	6194	37.5	307	20.4	
Male	20208	69.7	8672	79.3	10341	62.5	1195	79.6	
Mean age, years (±SD)	77.6±7.1		79.0±7.1		77.2±6.9		71.9±6.2		<0.001
Age group									<0.001
65–74	10994	37.9	3245	29.7	6639	40.1	1110	73.9	
74–84	13439	46.4	5514	50.4	7603	46.0	322	21.4	
85+	4545	15.7	2182	19.9	2293	13.9	70	4.7	
CCIS									<0.001
0 or 1	12792	44.1	5079	46.4	7056	42.7	657	43.7	
2	3813	13.2	1484	13.6	2152	13.0	177	11.8	
3	2736	9.4	990	9.0	1637	9.9	109	7.3	
4	9637	33.3	3388	31.0	5690	34.4	559	37.2	
Cancer group									<0.001
I	464	1.6	239	2.2	210	1.3	15	1.0	
II	811	2.8	364	3.3	418	2.5	29	1.9	
III	5169	17.8	1778	16.3	3125	18.9	266	17.7	
IV	8192	28.3	2966	27.1	4736	28.6	490	32.6	
V	5341	18.4	2121	19.4	3001	18.1	219	14.6	
VI	7997	27.6	3084	28.2	4483	27.1	430	28.6	
VII	1004	3.5	389	3.6	562	3.4	53	3.5	
Post-diagnosis survival, months									0.073
≤6	14699	50.7	5617	51.3	8370	50.6	712	47.4	
6.01–12	6206	21.4	2292	20.9	3567	21.6	347	23.1	
12.01–24	5591	19.3	2113	19.4	3159	19.1	319	21.2	
>24.01	2482	8.6	919	8.4	1439	8.7	124	8.3	
Primary physician’s specialty									0.001
Oncologist	3798	13.1	1329	12.1	2259	13.7	210	14.0	
Other	25180	86.9	9612	87.9	14276	86.3	1292	86.0	
Hospital characteristics									<0.001
Medical center	15387	53.1	6277	57.4	8175	49.4	935	62.3	
Regional	11646	40.2	3970	36.3	7147	43.2	529	35.2	
District	1945	6.7	694	6.3	1213	7.3	38	2.5	
Caseload group									<0.001
High	11077	38.2	4042	36.9	6586	39.8	449	29.9	
Medium	9303	32.1	3133	28.6	5648	34.2	522	34.8	
Low	8598	29.7	3766	34.5	4301	26.0	531	35.4	
Urbanization									<0.001
Urban	4817	16.6	3291	30.1	975	5.9	551	36.7	
Suburban	10437	36.0	5443	49.7	4288	25.9	706	47.0	
Rural	13724	47.4	2207	20.2	11272	68.2	245	16.3	
Geographic Region									<0.001
Northern	12366	42.7	7017	64.1	4493	27.2	856	57.0	
Central	4731	16.3	1352	12.4	3159	19.1	220	14.6	
Southern	10601	36.6	2103	19.2	8113	49.0	385	25.6	
Eastern	1278	4.4	468	4.3	769	4.7	41	2.8	

Cancer group I: nonmetastatic germ-cell tumors and prostate cancer; II: metastatic germ-cell tumors and prostate cancer; III: nonmetastatic lung, liver, and pancreatic cancer; IV: metastatic lung, liver, and pancreatic cancer; V: all other nonmetastatic cancers; VI: all other metastatic cancers; and VII: hematologic malignancies.

SD, standard deviation.

**Figure 1:**
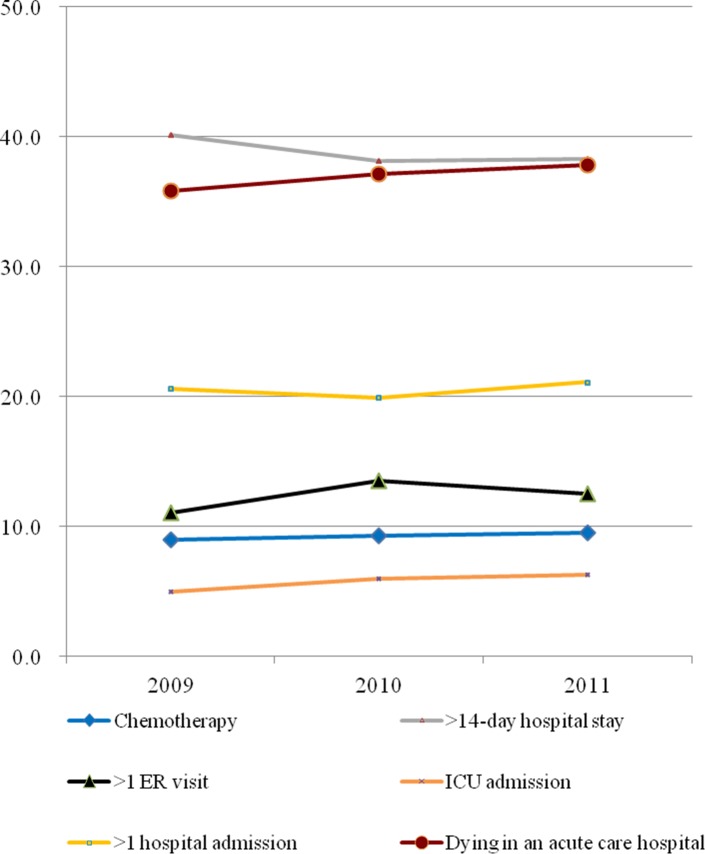
Trends for the six indicators of aggressive end-of-life care for Taiwanese cancer patients age 65 years and above for the period 2009 to 2011. ER, emergency room; ICU, intensive care unit.

The distribution of indicators for aggressive EOL care is provided in Fig. 1. The indicators had an increasing trend from 2009 to 2011, except for hospital stay >14 days. The number of indicators of aggressive EOL care averaged 1.26±1.16 for all study subjects.

**Figure 2:**
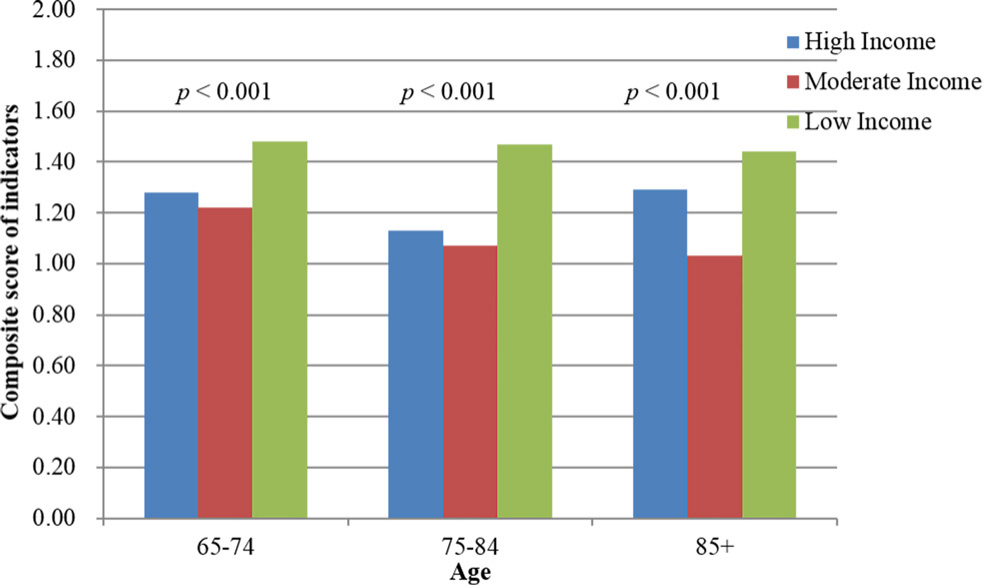
The impact of socioeconomic status (SES) on aggressiveness of end-of-life treatment by age.

Fig. 2 depicts the association of SES (individual income) and EOL care. Cancer decedents with low income were associated with having more aggressive EOL care. The aggressiveness of EOL care also declined with age. Hierarchical linear modeling using a random-intercept model revealed that, compared with decedents with low income, those with moderate (estimate-0.30, P<0.001) and high (estimate-0.27, P<0.001) income received less aggressive EOL care (Table 2). Male gender, being 65–75 years old, high CCIS, and post-diagnosis survival <6 months were associated with more aggressive EOL care. Furthermore, the aggressiveness of EOL treatment overall increased each year.

**Table 2:** Determinants of aggressive end-of-life care for Taiwanese cancer patients age 65 years and older, 2009–2011 by multivariate analysis using a random-intercept model (average indicator scores = 1.26±1.16).

**Parameter**	**Estimate**	**95%CI**	*p* **value**
Intercept	0.71	(0.51,0.91)	<0.001
SES			
Low	Reference		
Moderate	-0.30	(-0.33, -0.27)	<0.001
High	-0.27	(-0.33, -0.20)	<0.001
Gender			
Female	Reference		
Male	0.10	(0.07,0.13)	<0.001
Age group			
65–74	Reference		
75–84	-0.09	(-0.11, -0.06)	<0.001
85+	-0.10	(-0.14, -0.06)	<0.001
Charlson Comorbidity Index Score			
0 or 1	Reference		
2	0.21	(0.17,0.25)	<0.001
3	0.21	(0.17,0.26)	<0.001
≧4	0.26	(0.23,0.29)	<0.001
Cancer group			
I	Reference		
II	0.41	(0.28,0.54)	<0.001
III	0.37	(0.26,0.48)	<0.001
IV	0.60	(0.49,0.70)	<0.001
V	0.41	(0.30,0.52)	<0.001
VI	0.72	(0.61,0.83)	<0.001
VII	0.35	(0.22,0.48)	<0.001
Post-diagnosis survival, months			
≤6	Reference		
6.01–12	-0.07	(-0.10, -0.03)	<0.001
12.01–24	-0.11	(-0.15, -0.08)	<0.001
>24	-0.09	(-0.14, -0.08)	0.001
Primary physician’s specialty			
Other	Reference		
Oncologist	0.004	(-0.04,0.05)	0.841
Hospital characteristics			
District	Reference		
Medical center	0.02	(-0.10,0.14)	0.751
Regional	0.05	(-0.02,0.13)	0.180
Caseload group			
High	Reference		
Moderate	0.06	(-0.07,0.20)	0.327
Low	0.03	(-0.12,0.17)	0.672
Urbanization			
Urban	Reference		
Suburban	-0.02	(-0.06,0.02)	0.372
Rural	-0.02	(-0.07,0.03)	0.422
Geographic Region			
Northern	Reference		
Central	-0.03	(-0.08,0.03)	0.371
Southern	0.02	(-0.03,0.06)	0.459
Eastern	0.11	(0.02,0.20)	0.021
Year			
2009	Reference		
2010	0.06	(0.03,0.10)	<0.001
2011	0.08	(0.05,0.11)	<0.001

Cancer group I: nonmetastatic germ-cell tumors and prostate cancer; II: metastatic germ-cell tumors and prostate cancer; III: nonmetastatic lung, liver, and pancreatic cancer; IV: metastatic lung, liver, and pancreatic cancer; V: all other nonmetastatic cancers; VI: all other metastatic cancers; and VII: hematologic malignancies.

SES, socioeconomic status; EC, enrollee category. SD, standard deviation.

Compared to nonmetastatic germ-cell tumors and prostate cancer, decedents with cancer of poor prognosis (such as pancreatic, lung, and liver cancer) received more aggressive EOL care (Table 2). Decedents with distant metastasis cancer received more aggressive EOL care than those without metastasis.

**Figure 3:**
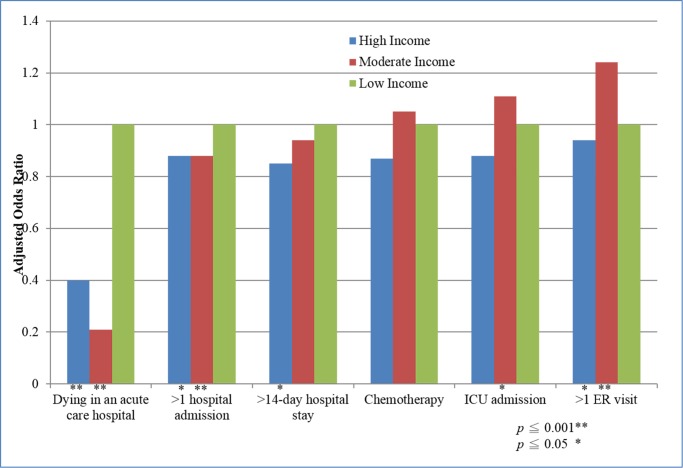
The differential effects of socioeconomic status (SES) on indicators of aggressive end-of-life care.

We further examined the association of each type of aggressive EOL care and income. Multilevel logistic regression analysis revealed that older cancer decedents with low income were more likely to stay in the hospital >14 days and to die in an acute hospital (Table 3 and Fig. 3). By contrast, older cancer decedents with moderate or high income visited the ER more than once and were admitted to the ICU more frequently than those with low income.

**Table 3:** Effects on SES categories on aggressive indicators of EOL care by multilevel logistic regression in older patients with cancer.

	Adjusted*
**Variable**	**OR**	**(95% CI)**	**P value**
**Dying in an acute care hospital**			
SES Low			
SES Moderate	0.26	0.24–0.27	<0.001
SES High	0.50	0.49–0.52	<0.001
**>1 hospital admission**			
SES Low	1		
SES Moderate	0.89	0.86–0.92	<.0001
SES High	0.93	0.90–0.96	0.04
**>14-day hospital stay**			
SES Low	1		
SES Moderate	0.94	0.92–0.97	0.03
SES High	0.96	0.94–1.00	0.20
**Chemotherapy**			
SES Low	1		
SES Moderate	1.06	1.00–1.11	0.15
SES High	1.00	0.95–1.05	0.90
**ICU admission**			
SES Low	1		
SES Moderate	1.17	1.11–1.25	0.005
SES High	1.06	1.00–1.13	0.31
**>1 ER visit**			
SES Low	1		
SES Moderate	1.23	1.18–1.28	<.0001
SES High	1.10	1.05–1.14	0.01

* Adjusted for patient age, gender, hospital spending index, Charlson Comorbidity Index Score, cancer group, primary physician’s specialty, post-diagnosis survival, hospital characteristics, hospital caseload, urbanization and geographic region.

SES, socioeconomic status; EOL, end-of-life; OR, odds ratio; CI, confidence interval; ER, emergency department; ICU, Intensive care unit;

## DISCUSSION

This study found that low individual income was associated with more aggressive EOL care in older cancer decedents in Taiwan. There was the greater tendency of older decedents with low income to die in the acute care hospital compared to more affluent decedents. Income was found to have differential effects on different indicators of aggressive EOL care. This difference by type of treatment may explain the disparities in results between studies. These results have implications for public health providers, who should offer hospice care to older cancer decedents with low income, to reduce the aggressiveness of the EOL care they receive and lessen the financial and emotional burden generated by such futile treatment.

The strength of our study is that it is a population-based observation study with abundant patient numbers to mitigate the effect of minor confounding factors. The Taiwan Health Insurance Program has covered approximately 99% of island residents for decades, and the validity of the dataset has been confirmed. We observed an influence of individual income on aggressiveness of EOL care in older cancer decedents, and further determined the effect of specific treatments on aggressive EOL care. To our knowledge, no previous studies have done this.

Determinants of place of death for terminal cancer decedents are complex. Factors such as age, gender, ethnicity, functional status, family support, personal and family preferences, hospice home visits, and details of the health care system all affect the choices of these decedents [23–27]. Taylor et al. revealed that decedents who die in an aged/residential care facility are more likely to be poorer than those who die elsewhere [28]. Cohen et al. found that education beyond high school was associated with greater likelihood of dying at home for cancer decedents living in Belgium, Italy, and Norway [27]. Motiwala et al. also showed that higher SES was associated with a slightly greater probability of dying at home [29]. Our study found that cancer decedents with low individual income were more likely than wealthier decedents to die in an acute care hospital, itself a major source of aggressive EOL treatment.

Our study revealed that male gender, high CCSI score, post-diagnosis survival <6 months, living in an urban area, and living in the northern region of Taiwan are associated with more aggressive EOL care. Other studies have already shown a relationship of male gender and post-diagnosis survival <6 months with more aggressive EOL treatment [15, 30, 31]. However, our findings differ from other studies in some respects. Thi et al. found that living in a rural area was associated with more aggressive EOL treatment in Canada [22]. But Lin et al. demonstrated increased hospice care in rural decedents over urban decedents in Taiwan [32]. This differential distribution of hospice care may explain why urban decedents received more aggressive EOL care in this study, since hospice care may reduce the incidence of aggressive EOL care [33].

In Sweden, Randén et al. found that having a high level of education was associated with more chemotherapy use [34]. Among older melanoma decedents, those residing in poorer SES areas were less likely to receive chemotherapy [35]. In decedents with non-small cell lung cancer, Saito et al. found no additional survival benefit from continuing chemotherapy within 14 days of death. In addition, continuing chemotherapy has been associated with a decreased likelihood of receiving hospice care [36]. In our study, SES had little differential effect on whether older decedents diagnosed with cancer continued chemotherapy.

In asthma decedents, lower SES was associated with higher odds of asthma-related ER/urgent care visits [37]. Hu et al. showed that geographical region of residence had a strong association with multiple ER visits in decedents with colorectal cancer in Alberta, Canada [38]. Our study showed that older cancer decedents with low SES have slightly lower likelihood of visiting the ER more than once, compared to more affluent decedents. The co-pay charge for an ER visit ($150) may deter low SES decedents from utilizing such care.

Previous studies have found that both low patient SES and low hospital area socioeconomic profile are associated with longer length of stay [39]. Hollowell et al. found that socioeconomically deprived decedents are more likely to remain in the hospital without morbidity following total knee replacement [40]. In contrast to these findings, this study found no significant difference in length of stay >14 days by SES in older decedents with cancer. It may be that decedents with terminal conditions view hospitalization differently or that their doctors recommend hospital stays differently than is the case with other types of conditions.

One limitation of the present study is that the cancer diagnosis and comorbidities were collected from the National Health Insurance claims using ICD-9 codes. While no administrative dataset is perfect, the National Health Insurance Bureau in Taiwan does randomly review charts and interview decedents to spot-verify the accuracy of diagnosis. Furthermore, some diseases have been validated in the NHIRD [41]. The second limitation is that we gave the same weight to each indicator of aggressive EOL care. Decedents from different cultures and societies may not consider such factors as of equal weight in making decisions about care. Given the robustness of the evidence and the statistical analysis in this study, these limitations are unlikely to compromise the validity of our results.

This study showed that older cancer decedents with low individual income were more likely to receive aggressive EOL care than those with high or medium income. Dying in an acute care hospital was the main factor related to this difference. We also found that the aggressiveness of EOL care for older decedents with cancer increased slowly over the past few years. Public health providers should be encouraged to educate their older cancer decedents on their disease prognosis and the benefits of hospice care, particularly when treating decedents with low income. Such strategies may reduce the rate of aggressive, but futile, EOL care. This reduction may in turn reduce the demand on staff, the emotional toll on decedents and their families, and the financial burden on the healthcare system.
